# Monitoring land degradation by soil salinity using Sentinel-2 satellite data and GIS techniques: A case study of Sabkhat Ghuwaymid, Saudi Arabia

**DOI:** 10.1371/journal.pone.0348799

**Published:** 2026-05-13

**Authors:** Ahmed Alneami, Abdullah Almuthibi, Merai Alqahtani, Ahmed A. Alameen, Shahd Alahmadi, Faisal Alharbi, Safiah Almutairi, Mohamed Alshafaey

**Affiliations:** General Administration of Technology and Digital Transformation, National Center for Vegetation Cover Development and Combating Desertification (NCVC), Riyadh, Saudi Arabia; Indian Institute of Technology Jammu, INDIA

## Abstract

Soil salinity drives land degradation and vegetation loss, posing a significant challenge for agriculture and global efforts to combat desertification. Enhancing land degradation monitoring methodologies is essential and aligns with the mission of the United Nations Convention to Combat Desertification (UNCCD), which aims to promote sustainable land management. In this context, this study aimed to develop soil salinity prediction models (SSPMs) for monitoring land degradation caused by soil salinity using Sentinel-2 (S2) satellite data and geographic information system (GIS) techniques. An area of 17.5 km^2^ (1750 ha) of Sabkhat Ghuwaymid, located in the Al-Qassim region of Saudi Arabia, was delineated as the study area for developing SSPMs. A total of 118 soil samples were collected using a grid sampling method between February 19 and 22, 2024. The collected samples were analysed for texture, acidity (pH: 6.97–8.61), total dissolved solids (TDS: 4.25–62.60 g L^-1^), and electrical conductivity (EC: 8.49–125.23 dS m^-1^) as an indicator of soil salinity. Soil analyses were performed using a hydrometer technique and Ohaus pH and EC meters. A promising model for predicting and mapping soil salinity was developed using S2 satellite imagery in combination with stepwise multiple linear regression (SMLR) analysis. The developed model demonstrated a correlation coefficient (R^2^) of 0.61 during development and 0.66 during validation, with a P-value < 0.05, and an RMSE of 12%. These results indicate that S2-based SSPMs provide a reliable and cost-effective approach for monitoring soil salinity, thereby supporting sustainable land management in arid environments.

## Introduction

The term “Sabkha” refers to low-lying and flat lands that are exposed to high rates of water evaporation, leaving behind various salts with different chemical compositions, which ultimately form a salt layer that forms the solid crust of the Sabkha [1]. The formation and thickness of the Sabkha are attributed to a combination of geological, hydrological, and climatic factors, including temperature, humidity, rainfall rate, and salinity levels [1]. Sabkha soil is found in many parts of the world, including the Arabian Gulf [2], North Africa [3], Sudan [4], Mexico [4], Australia [5], and the United States of America [6]. Globally, Sabkhas are estimated to cover approximately 30% of the Earth’s land surface [4].

Salinity, one of the most notable characteristics of Sabkha soil, is a crucial factor that influences nutrient availability, land production, and plant-soil interactions [7,8]. According to Corwin and Lesch [9], more than 50% (approximately 250 million hectares; m ha) of the world’s irrigated land is adversely affected by soil salinity, with about 20 m ha experiencing severe degradation. On the other hand, efforts to protect and repair salinity-affected lands have been prompted by the negative impacts of salinity on soil fertility, agricultural productivity, and afforestation initiatives [10].

Soil salinity is commonly assessed through electrical conductivity (EC) of the soil extract, expressed in terms of mS cm^-1^ or dS m^-1^, which is directly proportional to the concentration of dissolved salts in the soil solution [11]. Monitoring and identifying soil salinity are essential for improving vegetation cover development and combating desertification. Therefore, using advanced technology represented in remotely sensed data extracted from satellite imagery such as Sentinel-2, Landsat 7 and 8, Spot, and Worldview provides a unique solution for tracking soil salinity status at different spatial scales [12–14]. Among these, Sentinel-2 (S2) is a widely recognised and freely accessible source of multispectral data, offering 12 bands with spatial resolutions ranging from 10 to 60 m across the visible (VIS), near-infrared (NIR), and shortwave infrared (SWIR) regions. With a revisit time of five days [15], S2 imagery enables continuous monitoring of the Earth’s surface, making it a valuable tool for enhancing land degradation monitoring methodologies [14,16].

The pros of using advanced technologies represented in S2 imagery are numerous, particularly in terms of efficiency, accuracy, and cost-effectiveness. In contrast, conventional techniques, including field surveys and laboratory analyses, are inevitable, but are often time-consuming, costly, and labour-intensive [17]. For monitoring soil salinity, several indicators have been utilized, primarily based on the spectral behaviour of soils captured in multispectral imagery [18]. However, employing remote sensing (RS) techniques, two main categories of salinity indicators are commonly used: (i) direct indicators, such as the salinity index (SI), normalized difference salinity index (NDSI), and soil brightness index (BI); and (ii) indirect indicators, such as the normalized difference vegetation index (NDVI), enhanced vegetation index (EVI), soil-adjusted vegetation index (SAVI), and green difference vegetation index (GDVI) [19–22].

Leveraging the advantages of RS technology, numerous researchers have developed soil salinity prediction models (SSPMs) to monitor and assess soil salinity, enhance salinity mapping, enable more accurate prediction of salinity dynamics, and support land degradation monitoring, particularly in arid and semi-arid environments where field-based surveys are logistically challenging [8,12,14]. In such regions, regression-based SSPMs have been widely adopted due to their robustness under conditions of sparse vegetation, high surface reflectance, and strong salinity gradients [8,23]. Previous studies conducted in dryland environments have shown that linear and multivariate regression models can achieve reliable predictive performance by capturing the complex interactions among soil salinity, soil moisture, texture, and topographic factors [21,23,24].

In particular, stepwise multivariate linear regression (SMLR) provides a transparent and interpretable modelling framework that balances predictive reliability with practical applicability, making it well suited for soil salinity monitoring in saline and semi-arid environments [23,25]. Various approaches have been applied in soil salinity modelling, including regression-based methods and machine learning (ML) techniques [8,23–26]. These approaches offer notable advantages, such as capturing spatial variability across large areas [8,23], reducing the cost and effort associated with extensive field surveys [24], and enabling scalable and repeatable monitoring [25]. However, several limitations also exist, including spectral signals related to salinity may overlap with those associated with soil moisture and texture [24], some models are highly site-specific with limited transferability [23], and ML algorithms often require large calibration datasets and may be prone to overfitting [26]. Despite these limitations, regression-based approaches have consistently demonstrated reliable performance in soil salinity prediction studies [8,23,25]. Therefore, SMLR was adopted in this study to develop SSPMs.

Given the adverse effects of soil salinity on vegetation cover and afforestation initiatives led by the National Center for Vegetation Cover Development and Combating Desertification (NCVC) in Saudi Arabia, this study highlights the potential of RS techniques, particularly S2 imagery, for enhancing land degradation monitoring. Although RS based soil salinity assessment has been widely investigated at global and regional scales, most studies have focused on agricultural and coastal environments, whereas Sabkha environments in arid regions remain under-studied. In Saudi Arabia, existing research has primarily addressed soil salinity in cultivated lands and oases, with limited attention given to highly saline Sabkha environments and a lack of systematically developed and validated SSPMs. Within the broader global context, recent studies have demonstrated the potential of S2 imagery for salinity monitoring in arid and semi-arid regions [24]; however, model performance under conditions of extreme salinity, sparse vegetation cover, and complex micro-topography remains insufficiently explored.

Therefore, the main objective of this study is to develop SSPMs for monitoring land degradation driven by soil salinity using RS and geographic information systems (GIS) techniques in Sabkhat Ghuwaymid, Saudi Arabia, with potential applicability to other salt-affected environments. The central hypothesis is that S2-derived indices, when combined with regression-based modelling, can reliably predict soil salinity and provide appropriate salinity maps, especially in salt-affected environments. The specific objectives of this study are to: (i) analyse soil samples to determine salinity levels, (ii) extract and evaluate S2-derived vegetation and salinity indices, (iii) develop SMLR models linking soil salinity with spectral indices, and (iv) generate spatial salinity maps to support monitoring and land management. This study aligns with the objectives of both the NCVC and the United Nations Convention to Combat Desertification (UNCCD), while addressing a clear regional research gap and contributing to the global literature on land degradation monitoring in arid environments.

## Materials and methods

### Study area

**Fig 1:**
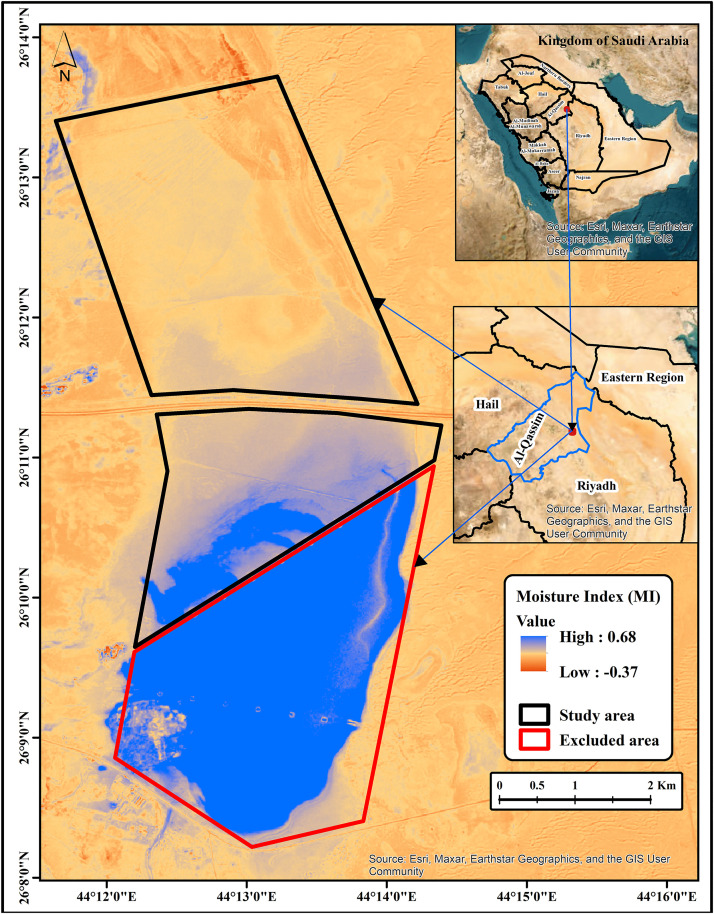
Moisture content in the study area (source: Esri, Maxar, Earthstar Geographics, and the GIS user community).

The fieldwork for this study was conducted in Sabkhat Ghuwaymid, located in the Al-Qassim region of Saudi Arabia, at coordinates 26°11′1.25″ N and 44°13′11.06″ E. The total study area covered 28 km^2^, of which 10.5 km^2^ exhibited high water content, as determined using the moisture index (MI). This portion (10.5 km^2^) was excluded due to the elevated MI values, which posed challenges during soil sampling. Consequently, an area of 17.5 km^2^ (1750 ha) was delineated to perform this study (Fig 1; source: Esri, Maxar, Earthstar Geographics, and the GIS user community). Field access and soil sampling were authorised by the National Center for Vegetation Cover Development and Combating Desertification (NCVC), Saudi Arabia, through the General Administration of Technology and Digital Transformation. Since the site is not located within a protected area and the activities were limited to non-destructive soil sampling, no additional permits were required.

The soil texture of the study area is predominantly silty loam, with negligible vegetation cover, except for sparse distributions of annual plants and shrubs in specific locations. This was confirmed through analysis of the normalized difference vegetation index (NDVI), as described in Equation 1 [27]. The study area is characterized by a semi-arid climate, with pronounced seasonal temperature variability and limited precipitation. Mean annual air temperatures range from approximately 10 °C during winter to up to 45 °C in summer, while mean annual rainfall ranges between 100 and 120 mm, with most precipitation occurring between November and March. Potential evapotranspiration rates substantially exceed precipitation throughout the year, resulting in a persistent moisture deficit that promotes salt accumulation in surface and near-surface soils. Hydrologically, the study area is influenced by a nearby water source that discharges into part of the site, contributing to localized soil moisture and influencing vegetation patterns (Fig 1). These climatic and hydrological conditions create a highly constrained environment for vegetation growth and strongly control soil salinity dynamics within the Sabkha system.


$$\text{NDVI}=\frac{NIR-RED}{NIR+RED}$$ (1)

Where NIR is the near infrared band and RED is the red band of the satellite image.

### Soil sampling strategy

**Fig 2:**
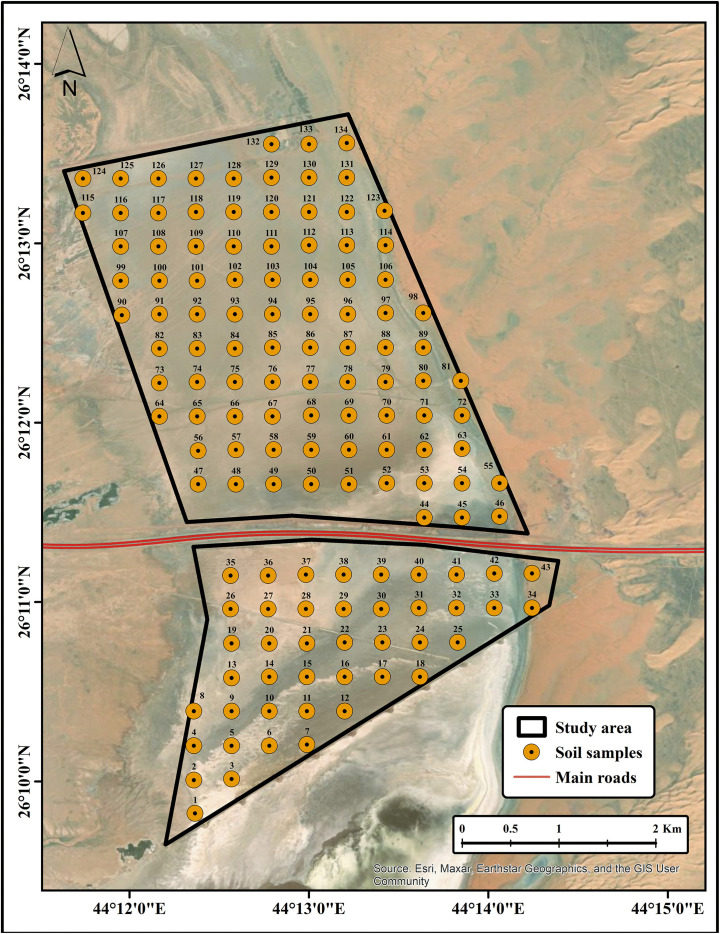
Soil sampling (source: Esri, Maxar, Earthstar Geographics, and the GIS user community).

Soil samples were collected from the study area to provide ground truth data for the development of soil salinity prediction models (SSPMs). Sampling was performed within the delineated 17.5 km^2^ area using a stratified grid sampling approach (350 * 350 m), generated using the Fishnet tool in ArcMap software (Version 10.8.2). A total of 118 soil samples were successfully collected out of 134; the remaining 16 samples could not be collected due to excessive water content (samples 3–7, 10–12, 15–18, and 22–25). Samples were collected from the topsoil section (15–20 cm depth) using a handheld GPS receiver (Garmin Montana 750i) to ensure accurate georeferencing during the period from February 19–22, 2024 (Fig 2; source: Esri, Maxar, Earthstar Geographics, and the GIS user community). The topsoil layer (15–20 cm) was selected for sampling, as it represents the zone most directly influenced by evaporation-driven salt accumulation and plant-soil interactions, and is critical for assessing soil salinity dynamics [28]. Moreover, this sampling depth is widely recommended in standard soil science protocols and has been commonly adopted in soil salinity studies to characterize surface salinity conditions and vegetation constraints in arid and semi-arid regions [28,29].

### Soil analysis

A total of 118 soil samples were collected, air-dried, and sieved through a 2 mm mesh. The samples were then mixed with distilled water at a 1:2 (soil: water) ratio [30] and carefully homogenised using an orbital shaker (Model: e-E51LD0403) before analysis. Soil parameters, including pH, total dissolved solids (TDS, g L ⁻ ¹), and electrical conductivity (EC, dS m ⁻ ¹), were measured from the prepared suspension. Measurements were replicated three times using an Ohaus pH meter (Model: AB 33PH) and an Ohaus EC meter (Model: AB 33EC) to ensure accuracy and reproducibility. Additionally, soil texture analysis was conducted using the hydrometer method, following the protocol established by the International Center for Agricultural Research in the Dry Areas (ICARDA). Documentation of field sampling and laboratory procedures is provided in S1 Fig.

### Remote sensing data

Remote sensing (RS) data were obtained from the Sentinel-2 (S2) satellite, specifically the Level-2A image scene with 11 bands (Tile ID: T38RMQ), as summarised in Table 1. The selected image corresponds to the field data collection period (February 22, 2024) and was downloaded from the Copernicus Open Access Hub (https://browser.dataspace.copernicus.eu/) which provides atmospherically corrected surface reflectance imagery (Table 2). Consequently, atmospheric correction and radiometric calibration were inherently applied prior to analysis through the Sen2Cor processing chain. Cloud masking was performed using the Scene Classification Layer (SCL) included in the Level-2A product, and pixels affected by clouds and cloud shadows were excluded from further analysis. For subsequent processing and the computation of soil salinity-related spectral indices, QGIS (Version 3.34.9) and SNAP software (Version 9.0.0), developed by the European Space Agency (ESA) for S2 image processing, were used to compute these indices. To ensure consistency among spectral bands, all S2 bands were resampled using the nearest neighbour method, thereby preserving the original spectral values.

**Table 1:** Spectral bands of S2 satellite image.

Spectral band	Region	Spatial resolution (m)	Wavelength (nm)
**B1**	Coastal aerosol	60	443
**B2**	Blue	10	490
**B3**	Green	10	560
**B4**	Red	10	665
**B5**	Red Edge 1	20	705
**B6**	Red Edge 2	20	740
**B7**	Red Edge 3	20	783
**B8**	Near infrared (NIR)	10	842
**B9**	Water vapor	60	945
**B11**	Shortwave infrared (SWIR 1)	20	1610
**B12**	Shortwave infrared (SWIR 2)	20	2190

**Table 2:** Specification of the S2 satellite image.

Product info.	Description	Product info.	Description
**Tile Id**	T38RMQ	Processed by	ESA
**Platform**	SENTINEL-2	Processing level	S2MSI2A
**Instrument**	MSI	Processor version	05.10
**Absolute orbit number**	45277	Processing date	February 22, 2024
**Acquisition mode**	INS-NOBS	Relative orbit number	92
**Cloud cover**	0.008396		

The downloaded S2 Fig was geo-rectified to the Universal Transverse Mercator (UTM) coordinate system using the World Geodetic System (WGS) 1984 datum, aligned with the north of UTM Zone 38, which corresponds to the study area. The region of interest (ROI) representing the delineated study area was then extracted for detailed analysis of spectral indices. These included salinity indices (SI), the soil brightness index (BI), the normalized difference salinity index (NDSI), and additional indices, as listed in Table 3. Such indices are not only suitable for capturing soil conditions within the current study area, but they are also widely recognised for their expected association with soil salinity in semi-arid and saline environments, as demonstrated in previous studies conducted under similar conditions [24,31,32].

**Table 3:** Spectral indices involved in the study.

#	Spectral indices	Formula	Reference
1	**Salinity index 1 (SI**_**1**_)	${\text{SI}}_{\text{1}}=\frac{\text{B2}}{\text{B4}}$	[33]
2	**Salinity index 2 (SI**_**2**_)	${\text{SI}}_{\text{2}}=\frac{\left(\text{B2}-\text{B4}\right)}{\left(\text{B2}+\text{B4}\right)}$	[22]
3	**Salinity index 3 (SI**_**3**_)	${\text{SI}}_{\text{4}}=\sqrt{\overset{\text{2}}{\underset{\text{3}}{(B}}+\overset{\text{2}}{\underset{\text{4}}{B}})}$	[33]
4	**Salinity index 4 (SI**_**4**_)	${\text{SI}}_{\text{4}}=\frac{\left(B\text{2}*B\text{4}\right)}{B\text{3}}$	[22]
5	**Salinity index 5 (SI**_**5**_)	${\text{SI}}_{\text{5}}=\frac{\text{B11}}{\text{B12}}$	[34]
6	**Normalized difference salinity index (NDSI)**	$\text{NDSI}=\frac{\left(\text{B4}-\text{B8}\right)}{\left(\text{B4}+\text{B8}\right)}$	[35]
7	**Simplified brightness index (SBI)**	($\text{SBI}=\sqrt{{(\text{B3}}^{\text{2}}+{\text{B8}}^{\text{2}}}$)	[19]
8	**Intensity**_**1**_ **(Int**_**1**_)	${\text{INT}}_{\text{1}}=\frac{\left(\text{B3}+\text{B4}\right)}{\text{2}}$	[24]
9	**Intensity**_**2**_ **(Int**_**2**_)	${\text{INT}}_{\text{2}}=\frac{\left(\text{B3}+\text{B4}+\text{B8}\right)}{\text{2}}$	[24]
10	**Soil moisture index (MI)**	$\text{MI }=\frac{\left(B\text{8}-B\text{1}\text{1}\right)}{\left(B\text{8}+B\text{1}\text{1}\right)}$	[36]
11	**Digital Elevation Model (DEM)**	Available Rater data (5 m resolution)	

### Soil salinity prediction models

**Fig 3:**
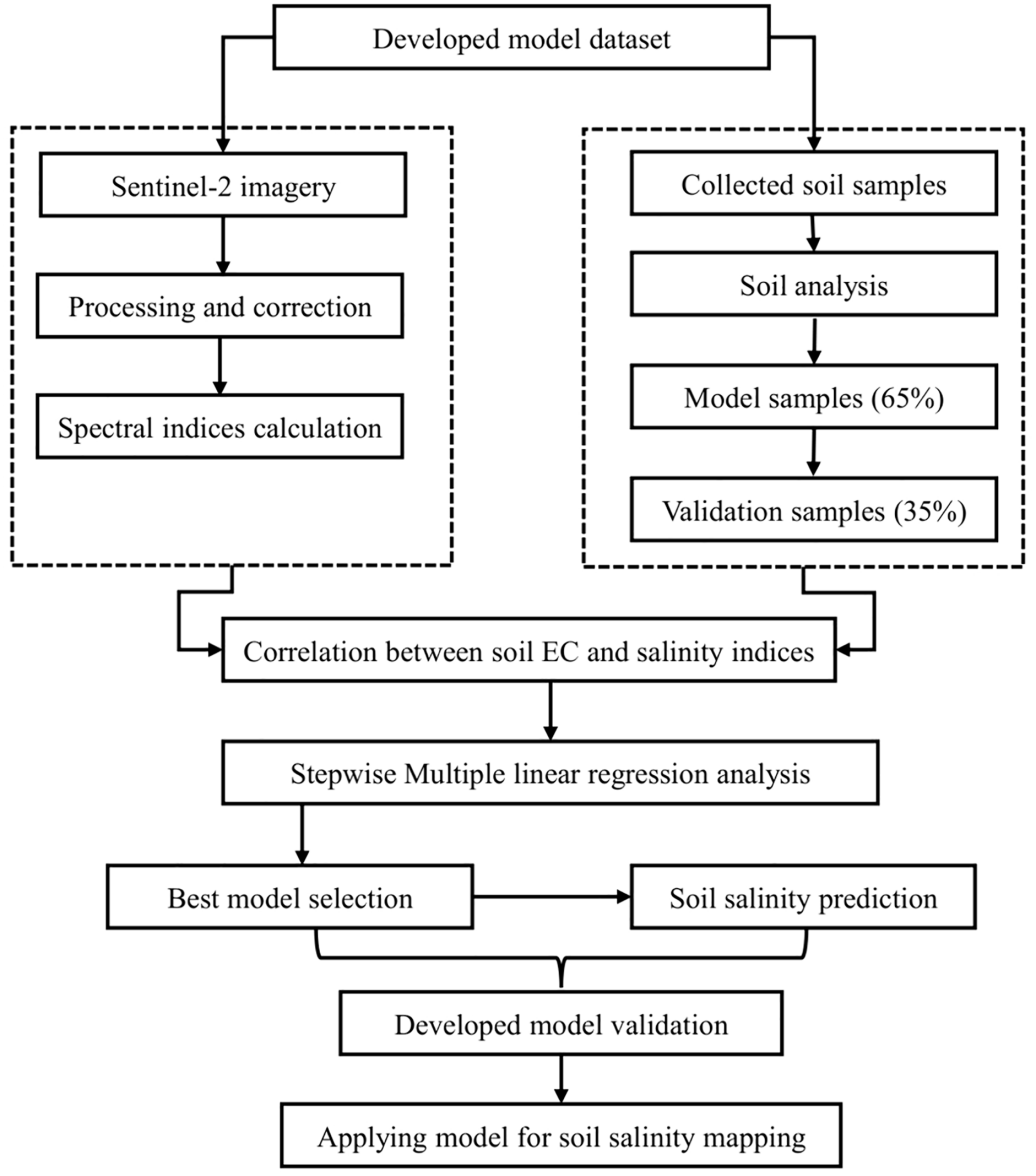
Methodology to develop the SSPMs.

Two types of datasets were employed and integrated to develop SSPMs for soil salinity mapping. The first dataset comprised RS data from S2 satellite, which was used to compute spectral indices associated with soil salinity. The second dataset consisted of ground-truth data (Measured soil EC), of which 65% was allocated for model development and the remaining 35% reserved for model validation. Stepwise multivariate linear regression (SMLR) analysis was conducted using SPSS statistical software (Version 20) to establish relationships between measured soil EC (dependent variable) and RS-derived indices (independent variables). The finalized SSPM was applied to map soil salinity across the study area using the Spatial Analyst tool (Inverse Distance Weighted, IDW) in ArcMap software (Version 10.8.2). IDW was selected as a practical and widely used interpolation method for generating spatial patterns of soil salinity. It provides a transparent and computationally efficient approach for spatial interpolation, making it suitable for effective soil salinity mapping and prediction [37]. The overall workflow for generating the SSPMs is illustrated in Fig 3.

### Assessment and validation of the developed salinity model

The effectiveness of the SSPMs was assessed using several statistical metrics, including the coefficient of correlation (R^2^), eigenvalue (EV), condition index (CI), and root mean square error (RMSE), as outlined in Equation 2 [38]. Subsequently, the selected model was validated using 35% of the collected data and then applied to predict and map soil salinity.


$$\text{RMSE}=\sqrt{\frac{\sum_{i=\text{1}}^{n}(y_{i}-\overset{*}{\underset{i}{y}}{)}^{\text{2}}}{n}}$$ (2)

Where, $y_{i}$ represents the measured soil EC (dS m^-1^), $\overset{*}{\underset{i}{y}}$ is the predicted soil EC (dS m^-1^), and n is the number of soil samples.

## Results and discussion

### Topographical and vegetation assessment

**Fig 4:**
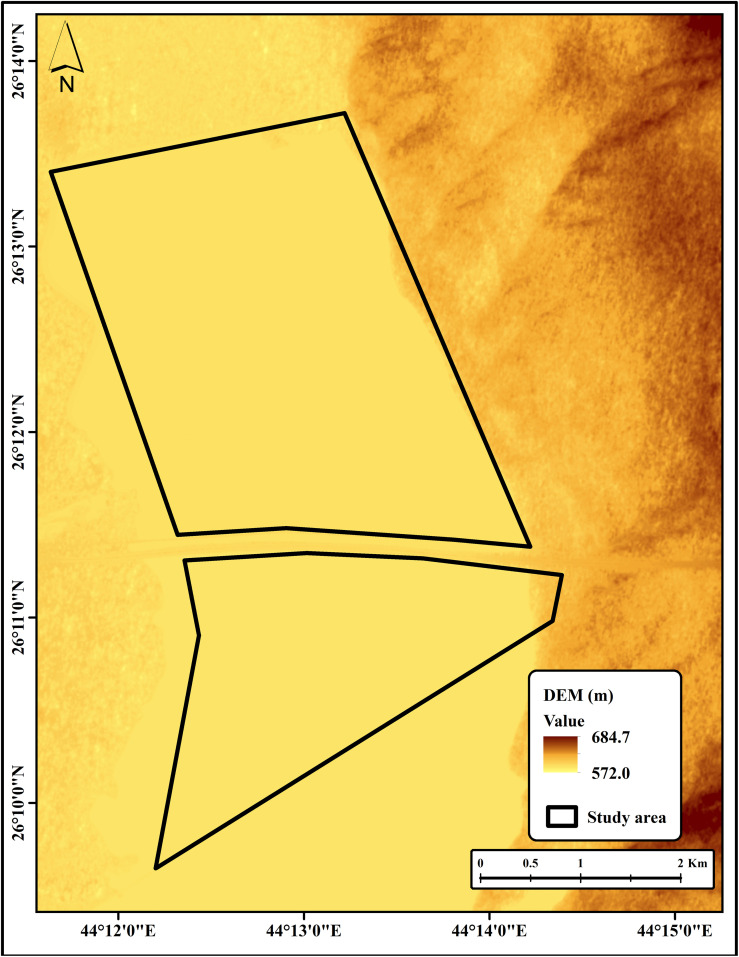
DEM of the study area.

**Fig 5:**
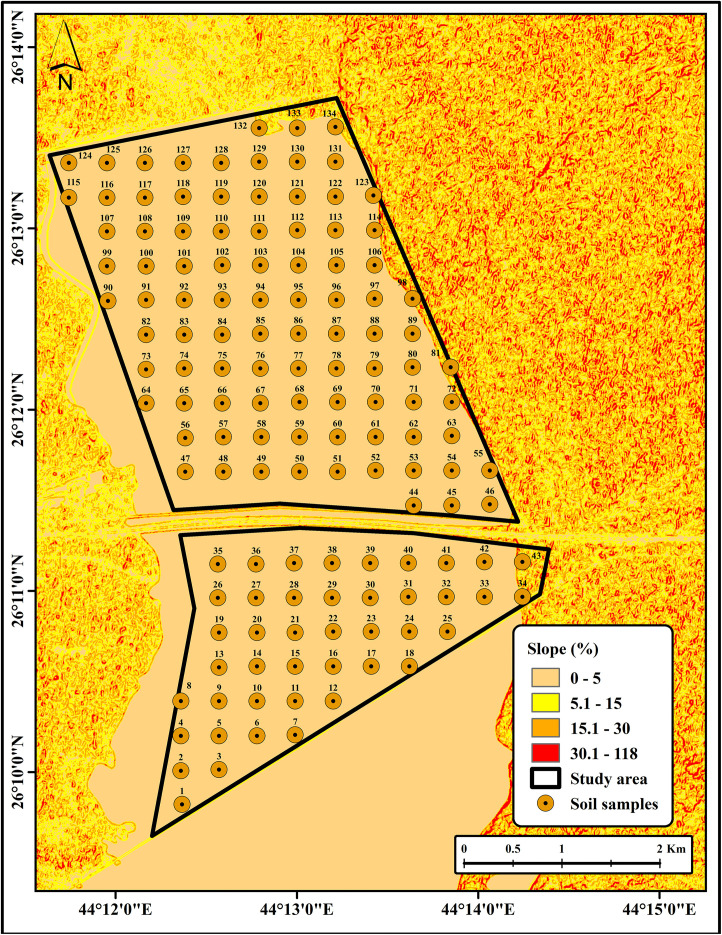
Slope of the study area.

The topography of the study area, as derived from the digital elevation model (DEM), revealed a predominantly flat surface with very gentle slopes ranging from 0 to 5%. However, the surrounding landscape is characterized by sand dunes exhibiting steep slopes of up to 118% (Figs 4 and 5). The presence of steep slopes around the perimeter likely influences hydrological processes, such as runoff and localized soil deposition, which in turn contribute to spatial heterogeneity in soil salinity. These findings suggest that micro-relief and slope gradients are primary controls of salt redistribution in arid soils, supporting previous observations that low-lying areas tend to accumulate higher concentrations of salts. Similar finding has been reported that slope gradients play a crucial role in redistributing salts through erosion and runoff processes, particularly in arid environments [39]. This underscores the importance of considering terrain effects when developing regional reclamation or management strategies.

**Fig 6:**
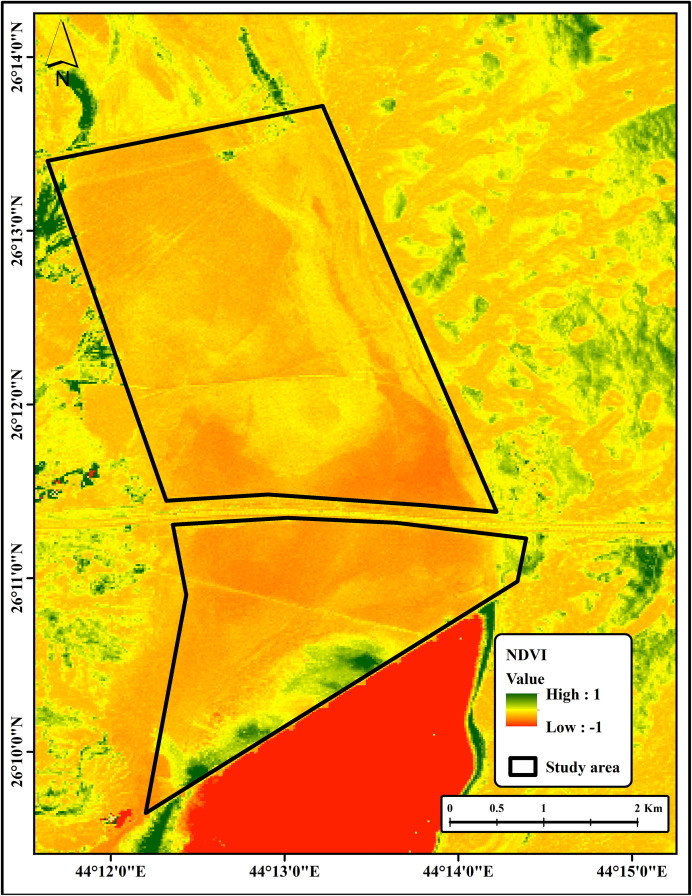
NDVI of the study area.

Additionally, the vegetation cover was assessed using the Normalized Difference Vegetation Index (NDVI); the assessment revealed that the high salinity levels of the study area substantially restrict plant growth. Consequently, vegetation was sparse, with only small shrubs and annual plants scattered across limited patches (Fig 6). This observation is consistent with previous studies, which have shown that elevated high levels of soil salinity impair plant physiological processes, reduce biomass, and lead to degraded vegetation cover in salt-affected soils [7,8].

### Soil properties

Table 4 presents the descriptive statistics of the measured soil parameters. The dominant soil texture in the study area was identified as silty loam. However, four samples (34, 43, 81, and 98), located near sand dunes at the study area boundary, exhibited a sandy texture distinct from the prevailing soil conditions. These samples were excluded from further analysis to maintain dataset homogeneity and ensure that the developed soil salinity prediction models (SSPMs) accurately represent the dominant soil matrix. A sensitivity check indicated that including these samples did not significantly improve model performance, confirming that their exclusion does not bias the overall results.

**Table 4:** Summary of measured soil properties.

Sample analysis	Soil Properties		
	pH	TDS (g L^-1^)	EC (dS m^-1^)
**Minimum**	6.97	4.25	8.49
**Maximum**	8.61	62.60	125.23
**Mean**	7.87	22.08	44.27
**Standard Error (SE)**	0.02	0.95	1.91

**Fig 7:**
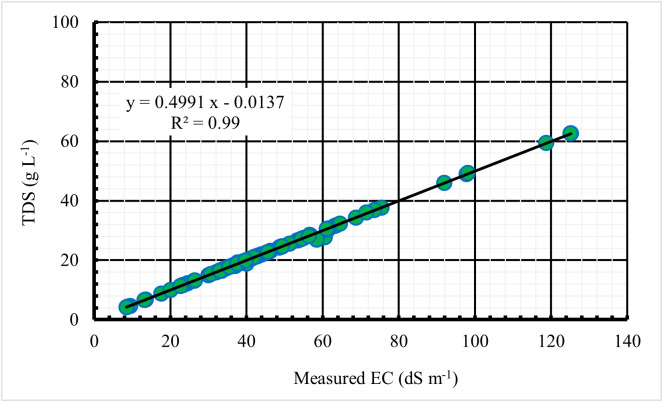
Relationship between soil EC and soil TDS.

The results indicated that the average soil moisture content of the study area was 18.3%. At the same time, the pH of the analysed samples (n = 114) ranged between 6.97 and 8.61, reflecting slightly neutral to moderately alkaline conditions. Soil electrical conductivity (EC) varied widely, from 8.49 to 125.23 dS m^-1^, while total dissolved solids (TDS) ranged from 4.25 to 62.60 g L^-1^. This wide variation in EC and TDS highlights the firm spatial heterogeneity of salinity within the study area, underscoring the importance of geospatial techniques for effective monitoring and mapping of salinity. A significant and robust linear correlation (R^2^ = 0.99, P < 0.05) was observed between soil EC and TDS (Fig 7), confirming that soil EC is a reliable proxy for assessing salinity levels. This finding corroborates previous studies that reported similarly strong EC-TDS relationships in saline soils [40,41]. Detailed soil analysis results are provided in S1 Table, supporting the observed spatial heterogeneity of soil salinity across the study area.

Additionally, soil salinity was classified based on EC values in terms of dS m^-1^ (Table 5), following the standard classification scheme of Morshed et al. [42]. According to this classification, the study area falls under the highly saline category, which poses severe limitations for agricultural production, impairs plant growth, and reduces crop yields. These results underscore the importance of spatial monitoring frameworks in guiding land management and soil reclamation strategies in salt-affected environments.

**Table 5:** Classification of soil salinity based on EC measurements.

Salinity class	EC (dS m^-1^)	Salinity class	EC (dS m^-1^)
**Non saline**	Less than 1	Medium salinity	4 - 6
**Very low salinity**	1 - 2	High salinity	6 - 8
**Low salinity**	2 - 4	Very high salinity	More than 8

### Analysis of remote sensing data

A total of 114 soil samples were used to develop soil salinity prediction models (SSPMs), with approximately 65% allocated for model training and 35% for validation. This proportion was selected to balance the need for sufficient data to develop a robust model, while reserving an adequate portion of independent data for reliable validation. Similar proportions (65−70% for training and 35−40% for validation) have been widely adopted in remote sensing and environmental modelling studies, demonstrating their suitability for predictive applications in heterogeneous environments [14,24,31,43]. The relationships between soil EC and Sentinel-2 (S2) derived data, including both spectral bands and spectral indices, provided valuable insights into their predictive capabilities [14,44]. The coefficient of correlation (R^2^) and associated P-values were used to assess the strength and significance of these relationships, as shown in Table 6.

**Table 6:** Relationship between soil EC and remote sensing data.

Spectral bands	Coefficient of correlation (R^2^)	P-value	Spectral indices	Coefficient of correlation (R^2^)	P-value
**B1**	0.422	0.000	SI_1_	0.446	0.000
**B2**	0.372	0.001	SI_2_	0.423	0.000
**B3**	0.303	0.005	SI_3_	0.423	0.000
**B4**	0.172	0.074	SI_4_	0.391	0.000
**B5**	0.163	0.086	SI_5_	0.681	0.000
**B6**	0.144	0.114	NDSI	0.305	0.005
**B7**	0.121	0.156	SBI	0.265	0.012
**B8**	0.099	0.203	INT_1_	0.267	0.012
**B9**	0.065	0.294	INT_2_	0.228	0.027
**B11**	− 0.373	0.001	MI	0.496	0.000
**B12**	− 0.556	0.000	DEM	− 0.681	0.000

Statistical significance is evaluated based on P-values (P < 0.05).

The spectral bands (B1 to B12) displayed varying levels of explanatory power for predicting soil EC. Among these, B1 (R^2^ = 0.422) and B2 (R^2^ = 0.372) exhibited the strongest positive correlation with soil EC, and both were statistically significant at P < 0.05. In contrast, bands B4-B9 showed weak correlations (R^2^ = 0.06–0.17, P = 0.07–0.29), underscoring their limited predictive utility of soil EC. Interestingly, the shortwave infrared (SWIR) bands (B11 and B12) exhibited statistically significant negative correlations with soil EC (R^2^ = − 0.373 and R^2^ = − 0.556, respectively; P ≤ 0.05). This implies that higher SWIR reflectance corresponds to lower soil salinity levels, which may be attributed to the sensitivity of SWIR wavelengths to soil moisture and salt crystallization patterns. Similar results were reported by Al-Gaadi et al. [24], Wang et al. [44], and Hihi et al. [45] when demonstrating the potential of SWIR bands in soil salinity monitoring and mapping using S2 imagery. Together, these results indicate that while visible and near-infrared (NIR) bands have limited potential for capturing soil EC, SWIR bands may provide practical information on soil chemical properties, particularly salinity.

Despite some significant relationships, the overall performance of individual bands remained weak, reflecting their limited ability to capture soil EC. This outcome aligns with previous studies carried out in arid and semi-arid regions, which reported that soil EC is influenced by complex interactions between soil texture, moisture, and vegetation cover rather than a single spectral response [14,21]. Consequently, this highlights the necessity of integrating multiple spectral features to enhance prediction accuracy.

Compared with individual bands, the studied spectral indices (SI_1_ to DEM-derived indices) outperformed the individual bands in terms of predictive strength. SI_5_ (R^2^ = 0.681) emerged as the best predictor, followed by MI (R^2^ = 0.496) and SI_2_ (R^2^ = 0.423), all statistically significant with P = 0.001. The predictive capability of SI_2_ and SI_5_ in predicting soil EC is consistent with findings by Solangi et al. [46] and Al-Gaadi et al. [24], who reported an R^2^ value of 0.93 and 0.60, respectively. The improved performance of these indices can be attributed to their integration with multiple bands, which helps mitigate noise and capture soil salinity signals more effectively than single-band approaches [33,47,48]. Furthermore, the DEM presented a relatively strong correlation with soil EC (R^2^ = − 0.681), suggesting that elevation has a significant influence on soil EC distribution, likely through underlying environmental gradients [39,49].

Overall, these findings demonstrate that spectral indices integrated with multivariate regression modelling provide a more effective framework for capturing soil EC than individual spectral bands. While single-band information may offer preliminary insights, especially in the SWIR range, incorporating spectral indices and environmental covariates, such as DEM, substantially improves predictive reliability. This highlights the importance of adopting integrated approaches that combine remote sensing indices with ancillary data to capture the complex spatial variability of soil EC.

### Accuracy of soil salinity prediction models

By utilizing stepwise multiple linear regression (SMLR) analysis [50], three SSPMs were effectively developed, as illustrated in Table 7. The progressive improvement from Model 1 to Model 3 demonstrates that incorporating topographic variables alongside spectral information considerably enhances prediction accuracy. Among the evaluated variables, spectral indices (SI_2_, SI_5_, and DEM) were consistently identified as the best predictors of soil EC.

**Table 7:** Summary of the developed soil salinity models.

Model	SSPMs Summary					
	Predictors	Model	R	R^2^	Adjusted R^2^	SE
**1**	SI_5_	EC = 96.739 * SI_5_–79.904	0.684	0.468	0.461	17.56
**2**	SI_2_, SI_5_	EC = 37.562 * SI_2_ + 89.532 *SI_5_–54.671	0.755	0.570	0.558	15.91
**3**	SI_2_, SI_5_, DEM	EC = 34.413 * SI_2_ + 55.942 *SI_5_–11.466 * DEM + 6694.389	0.779	0.606	0.589	15.33

The performance indicators used to assess the developed models include multiple statistical measures, such as the coefficient of correlation (R^2^), adjusted R^2^, standard error of the estimate (SE), significance level (P-value), eigenvalue (EV), and condition index (CI). These evaluation indicators have been widely applied in modelling soil properties such as salinity, organic carbon, and calcium carbonate content, providing a comprehensive assessment of predictive reliability [31,43]. Moreover, ANOVA results (Table 8) confirmed a highly significant relationship (P < 0.001) between the soil EC and the selected predictors. Detailed statistical diagnostics of the developed models are presented in S2 Tables, providing additional support for the robustness and validity of the modelling approach.

**Table 8:** ANOVA results of the developed soil salinity models.

ANOVA analysis						
Model		Sum of Squares	df	Mean Square	F	P-value
**1**	Regression	19036.51	1	19036.51	61.67	.000
	Residual	21607.61	70	308.68		
	Total	40644.13	71			
**2**	Regression	23172.14	2	11586.07	45.75	.000
	Residual	17471.99	69	253.21		
	Total	40644.13	71			
**3**	Regression	24646.20	3	8215.40	34.92	.000
	Residual	15997.92	68	235.26		
	Total	40644.13	71			

**Fig 8:**
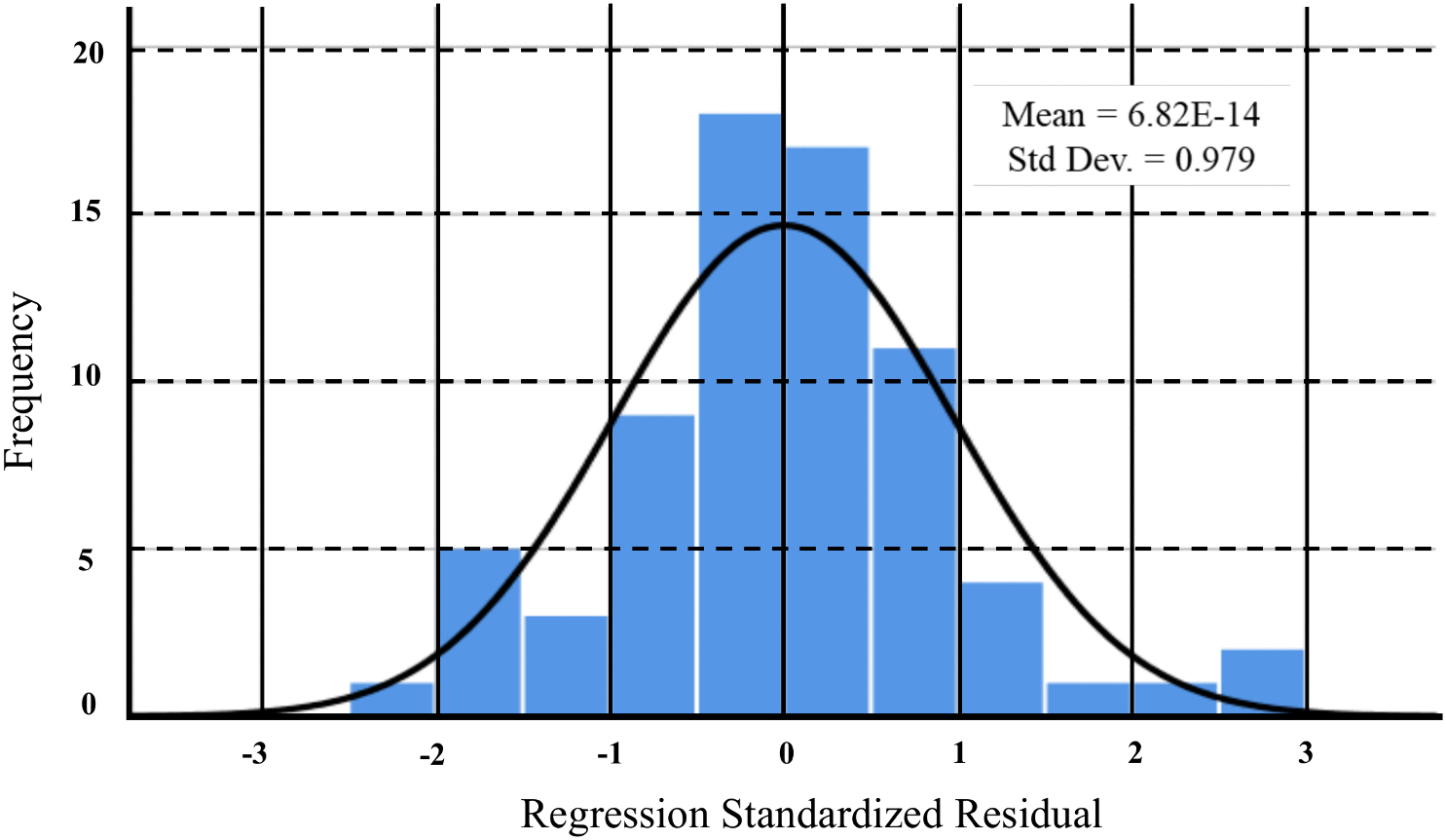
Histogram of the developed model.

Among the three developed models, Model 3 outperformed the others, achieving an R^2^ value of 0.61, an SE of 15.33, and a P < 0.001. Also, the residuals histogram of Model 3 (Fig 8) displayed a symmetric and normal distribution centred around zero, indicating unbiased and acceptable prediction capability of soil EC. Additionally, the collinearity diagnostics (EV and CI) confirmed the robustness of Model 3, with EV not close to zero and CI values less than 15 (Table 9), suggesting no multicollinearity issues among predictors.

**Table 9:** Collinearity diagnostics of the developed salinity models.

Collinearity diagnostics							
Model	Dimension	EV	CI	Variance Proportions			
				(Constant)	SI_2_	SI_5_	DEM
**1**	1	1.992	1	.00		.00	
	2	.008	15.43	1.00		1.00	
**2**	1	2.852	1	.00	0.02	.00	
	2	.140	4.51	.01	0.89	.02	
	3	.008	19.30	.99	0.09	.97	
**3**	1	3.837	1	.00	0.01	.00	.00
	2	.153	5.01	.00	0.90	.00	.00
	3	.011	19.10	.00	0.07	.39	.00
	4	2.261E-7	4119.06	1.00	0.02	.61	1.00

**Fig 9:**
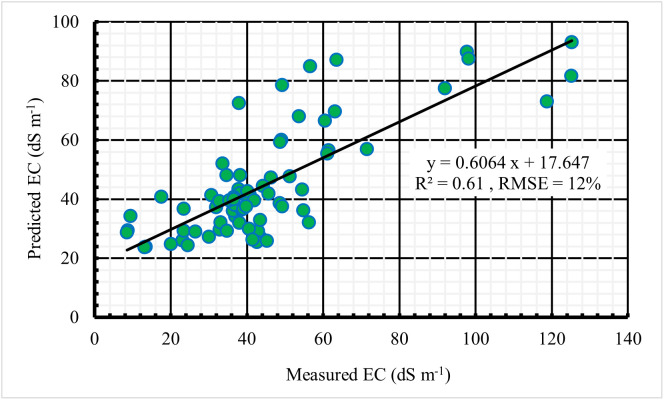
Relationship between measured and predicted soil EC.

**Fig 10:**
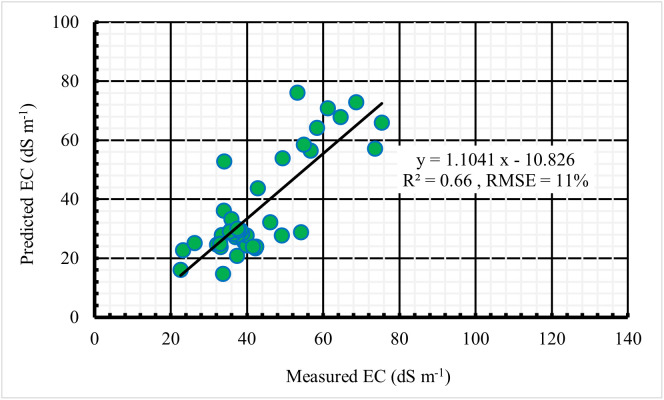
Validation of the developed model.

The relationship between measured soil EC and predicted soil EC using Model 3 is illustrated in Fig 9, with an R^2^ of 0.61 and an RMSE of 12%. Independent validation using 35% of the dataset showed slightly improved performance with an R^2^ of 0.66 (Fig 10), further confirming the model’s reliability. Achieving high 𝑅^2^ values at large spatial scales is challenging, as soil salinity is influenced by multiple interacting factors, including soil texture, moisture, and vegetation cover [14,21].

In this study, the moderate R^2^ values can be attributed to environmental heterogeneity typical of Sabkha landscapes. Although soil texture across the study area is relatively uniform, substantial spatial variability in salinity was observed, as reflected by the wide range of EC values and a high coefficient of variation. This variability is largely driven by differences in soil moisture and subtle microtopographic variations that influence evaporation and subsequent salt redistribution. As a result, salinity often occurs in patchy patterns over short distances, which are difficult to fully capture using medium-resolution satellite imagery, thereby weakening the correlation between spectral indices and soil salinity.

Consequently, while the model explains approximately 61–66% of the observed variability in soil salinity, a remaining portion (approximately 34–39%) remains unexplained. This residual variability reflects the influence of complex environmental factors that are not fully captured by the selected predictors. The relatively high RMSE observed in this study reflects the considerable spatial variability of soil salinity across the study area (coefficient of variation (CV) = 46%). This may introduce a degree of local uncertainty and potential misclassification, particularly in highly heterogeneous zones with abrupt salinity gradients. Such behavior is well documented in arid and semi-arid environments, where salinity distribution is influenced by micro-topography, groundwater dynamics, and localized depositional processes [51,52].

Comparable findings have been reported in arid environments. Sentinel-2-based models for predicting soil salinity have achieved R^2^ values ranging from 0.59 to 0.63 [31]. In a similar context, Al-Gaadi et al. [24] developed three prediction salinity models with R^2^ values between 0.44 and 0.62, accompanied by RMSE values of 10.2% − 14.3%. Hihi et al. [45] also reported a salinity prediction model with an R^2^ of 0.48 and an RMSE of 15.2%. Furthermore, Allbed et al. [52] developed a salinity model that achieved an R^2^ of 0.65 during model development but dropped to 0.34 during validation, with an RMSE of 19.4%. The elevated RMSE was attributed to a high CV value of 85.3%. Collectively, these results corroborate the findings of the present study and highlight the persistent challenges associated with capturing fine-scale soil salinity variability in highly heterogeneous landscapes. Nevertheless, by integrating Sentinel-2-derived spectral indices with topographic variables and regression analysis, the present study achieved slightly higher predictive accuracy (R^2^ = 0.61–0.66) compared to previous studies reporting R^2^ values of 0.44–0.65 [24,31,45,52]. These results highlight the robustness of this combined remote sensing and regression-based modelling approach for monitoring soil salinity in arid environments.

Several limitations of this study should be acknowledged. First, the spatial resolution of S2 imagery (10–20 m for key bands) may not capture fine-scale salinity variability in heterogeneous landscapes. Second, the SMLR models may oversimplify complex nonlinear interactions among soil properties, vegetation cover, and terrain factors. Third, the dataset is site-specific to Sabkhat Ghuwaymid; therefore, model parameters may require recalibration before application in other regions.

Despite these constraints, this study provides novel contributions to advancing soil salinity monitoring in salt-affected environments. It represents one of the first to comprehensive evaluations of S2-derived spectral and topographic predictors of soil salinity in Sabkhat Ghuwaymid, a highly saline and under-researched environment. By integrating field observations, remote sensing data, and regression-based modelling, as recommended in precision agriculture and land degradation studies [31,53,54], the study identifies key spectral indices and environmental variables relevant for semi-arid salt-affected areas. In particular, the integration of spectral salinity indices with topographic information (DEM) improves the ability to capture spatial variability in soil salinity across heterogeneous arid and semi-arid landscapes. This approach provides a transferable framework for soil salinity assessment that extends beyond traditional descriptive mapping and contributes to improved land degradation monitoring in arid and semi-arid regions. Consequently, the findings of this study help address a critical knowledge gap in both regional studies in Saudi Arabia and the broader global literature on soil salinity monitoring.

### Soil salinity mapping

**Fig 11:**
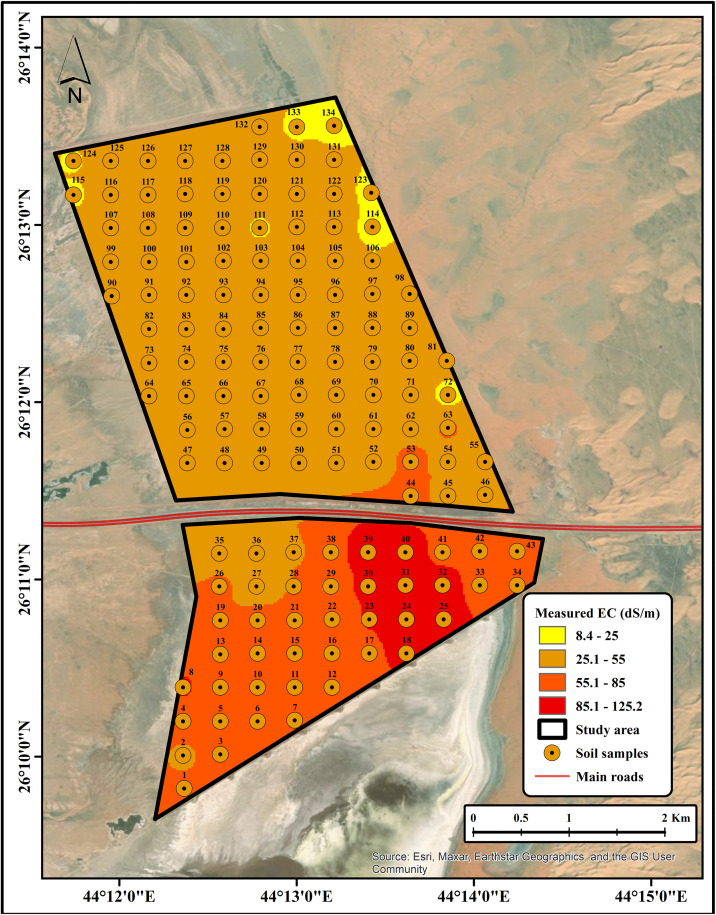
Measured EC map (source: Esri, Maxar, Earthstar Geographics, and the GIS user community).

**Fig 12:**
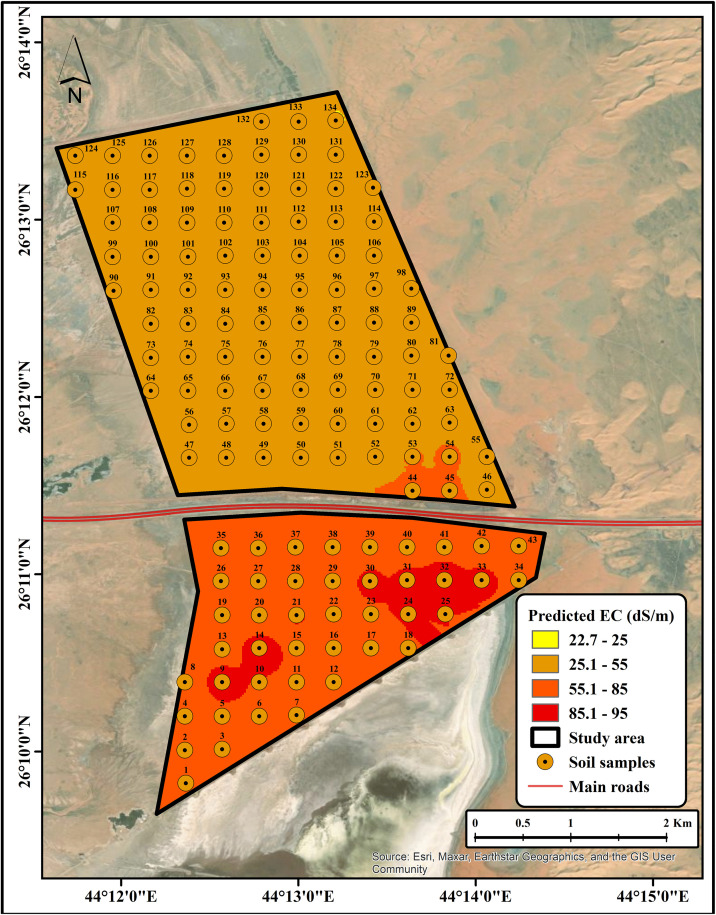
Predicted EC map (source: Esri, Maxar, Earthstar Geographics, and the GIS user community).

The development of SSPMs provides a practical approach for effective salinity mapping, which is essential for agricultural planning and resource management. The measured soil EC map (Fig 11; source: Esri, Maxar, Earthstar Geographics, and the GIS user community) captures actual salinity levels derived from collected soil samples, while the predicted map (Fig 12; source: Esri, Maxar, Earthstar Geographics, and the GIS user community) demonstrates the model’s capability to reproduce these spatial patterns using key predictors, including spectral indices and terrain variables. The close correspondence between measured and predicted maps highlights the model’s reliability, although minor discrepancies indicate areas where further refinement may be beneficial. Collectively, SSPMs provide a cost-effective and accurate tool for identifying and delineating salinity-affected areas, thereby supporting sustainable land management and precision agriculture practices [55,56]. This approach can be readily applied to similar arid and semi-arid environments, allowing decision-makers to prioritize areas for soil reclamation or alternative cropping strategies. Overall, the integration of remote sensing, field observations, and statistical modelling represents a robust framework for managing soil salinity in fragile ecosystems.

## Conclusions

This study highlights the critical role of remote sensing (RS), particularly Sentinel-2 (S2) satellite imagery, in advancing soil salinity monitoring to support sustainable land management. By integrating ground-truth data with RS-derived spectral indices, a soil salinity prediction model (SSPM) was developed and validated, achieving moderate and reliable predictive performance (R^²^ = 0.61 for development and 0.66 for validation; RMSE = 12%). Key findings indicated that spectral indices, particularly salinity index 2 (SI_2_), salinity index 5 (SI_5_), and soil moisture index (MI), outperform individual spectral bands in capturing complex salinity patterns, and the inclusion of Digital Elevation Model (DEM) improves model reliability.

These results demonstrate the efficiency, cost-effectiveness, and accuracy of satellite-based approaches, offering a practical tool for real-time monitoring and targeted interventions to combat land degradation. The developed SSPM has potential application in other salt-affected areas and can enhance sustainable agriculture and afforestation programmes. Despite its demonstrated effectiveness, the proposed approach has inherent limitations associated with the spatial resolution of S2 imagery, the potential oversimplification of complex nonlinear soil-environment interactions by regression-based modelling, and the site-specific nature of the derived model parameters. Future research could explore the integration of higher-resolution RS data and advanced machine learning (ML) techniques, such as Random Forest (RF) for modelling nonlinear relationships and variable interactions, Support Vector Machines (SVM) for handling high-dimensional feature spaces with limited training samples, and artificial neural networks (ANN) for capturing complex soil-spectral relationships. Such approaches could enhance predictive accuracy and support regional-scale applications in arid and semi-arid environments.

## Supporting information

S1 Fig Field and laboratory work. (DOCX)

S1 Table Soil analysis results. (XLSX)

S2 Tables Statistical analysis. (DOCX)
