# Supplementary material for: Monitoring land degradation by soil salinity using Sentinel-2 satellite data and GIS techniques: A case study of Sabkhat Ghuwaymid, Saudi Arabia
Source: PLoS One. 2026 May 13;21(5):e0348799. doi: 10.1371/journal.pone.0348799 (PMC13170892; doi:10.1371/journal.pone.0348799)
Supplement: S1 Fig — (DOCX) [file pone.0348799.s001.docx]

**Field and Lab work**


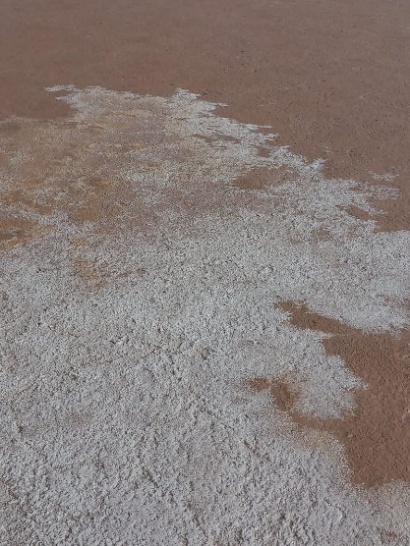

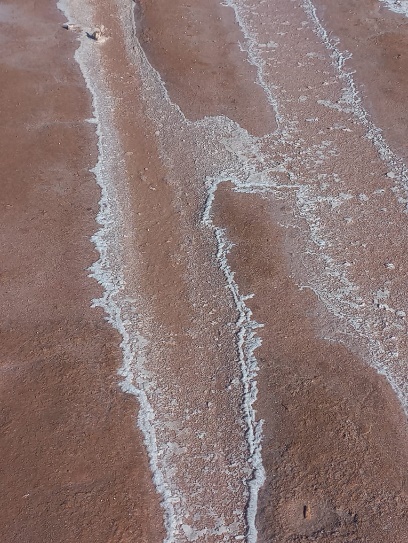

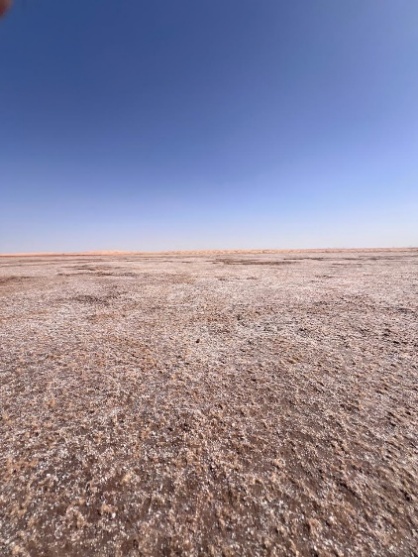


**Study area**


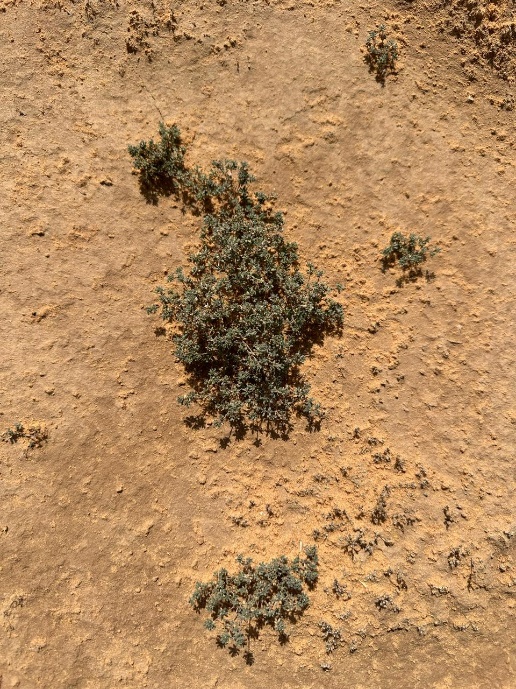

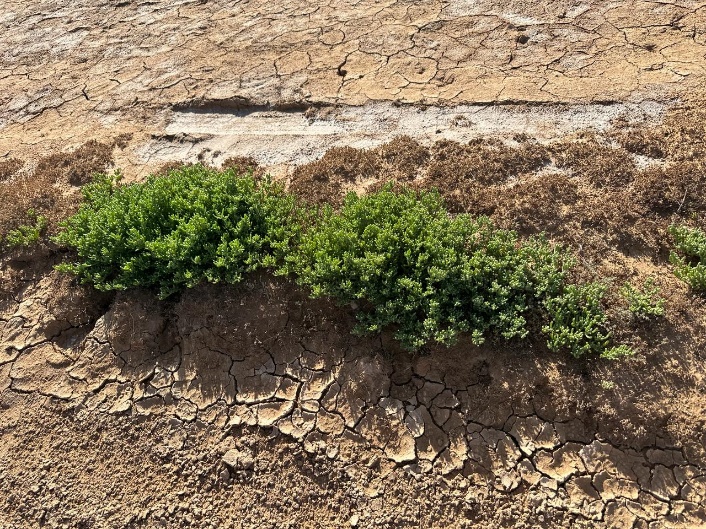


**Annuals and small shrubs in the study area**


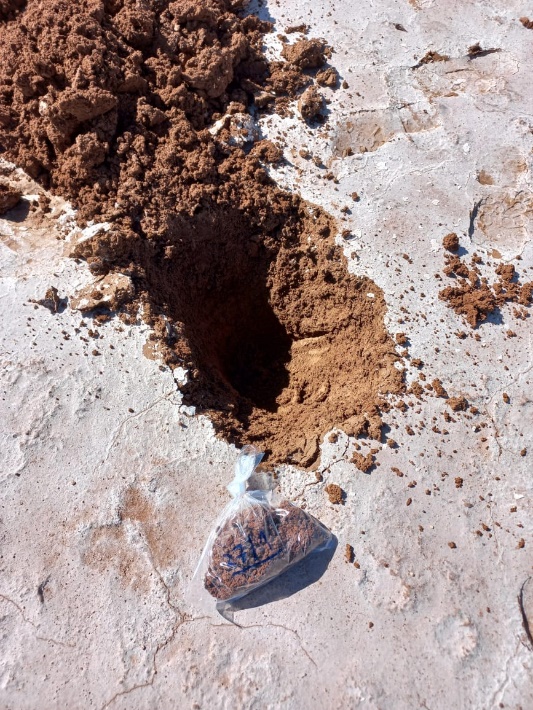


**Soil samples collection**


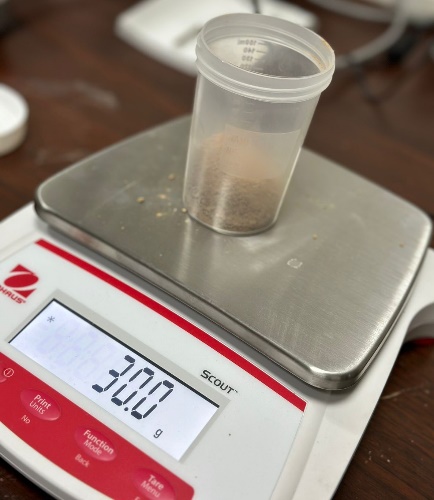

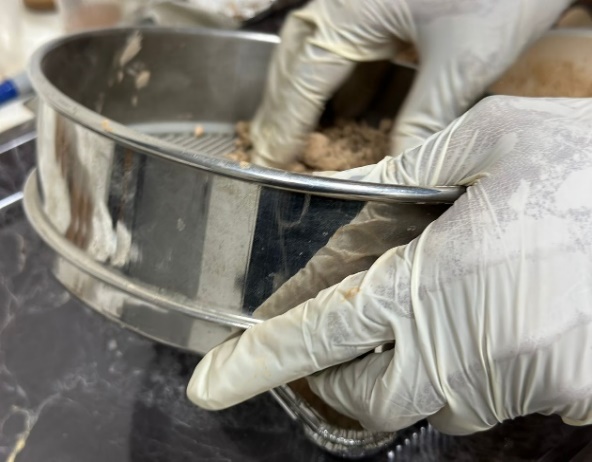

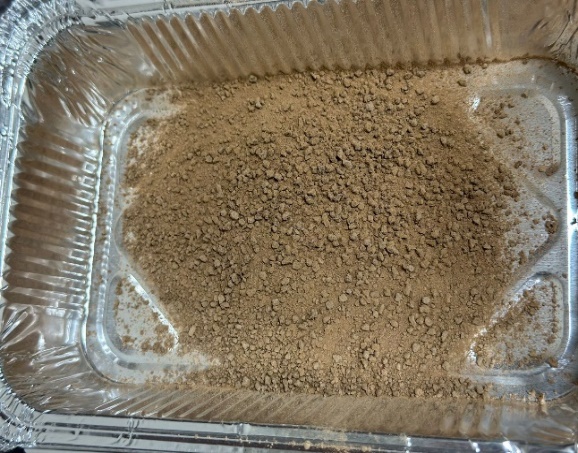


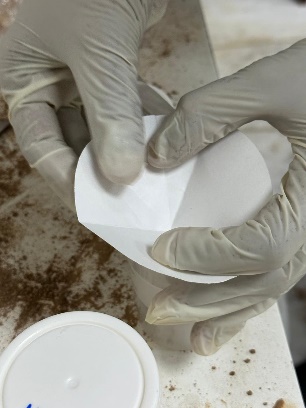


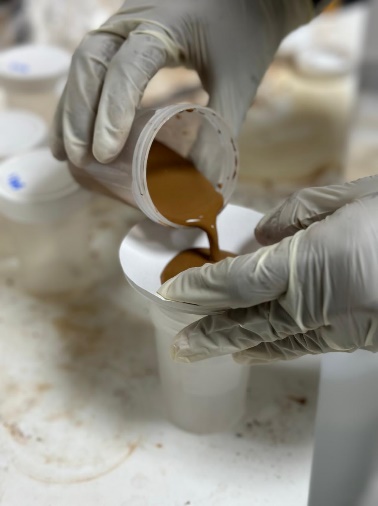


**lab analysis of soil samples**


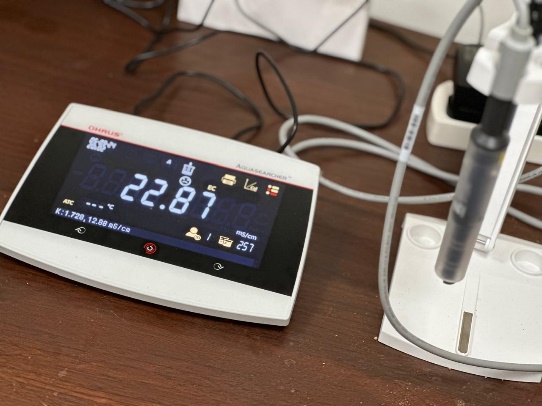

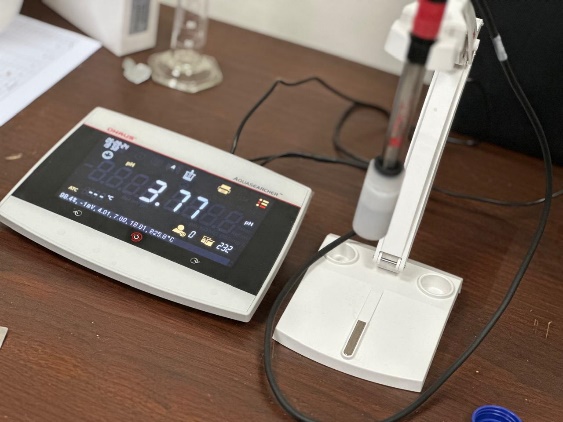


**Soil pH, EC, and TDS analysis (Ohaus meters)**


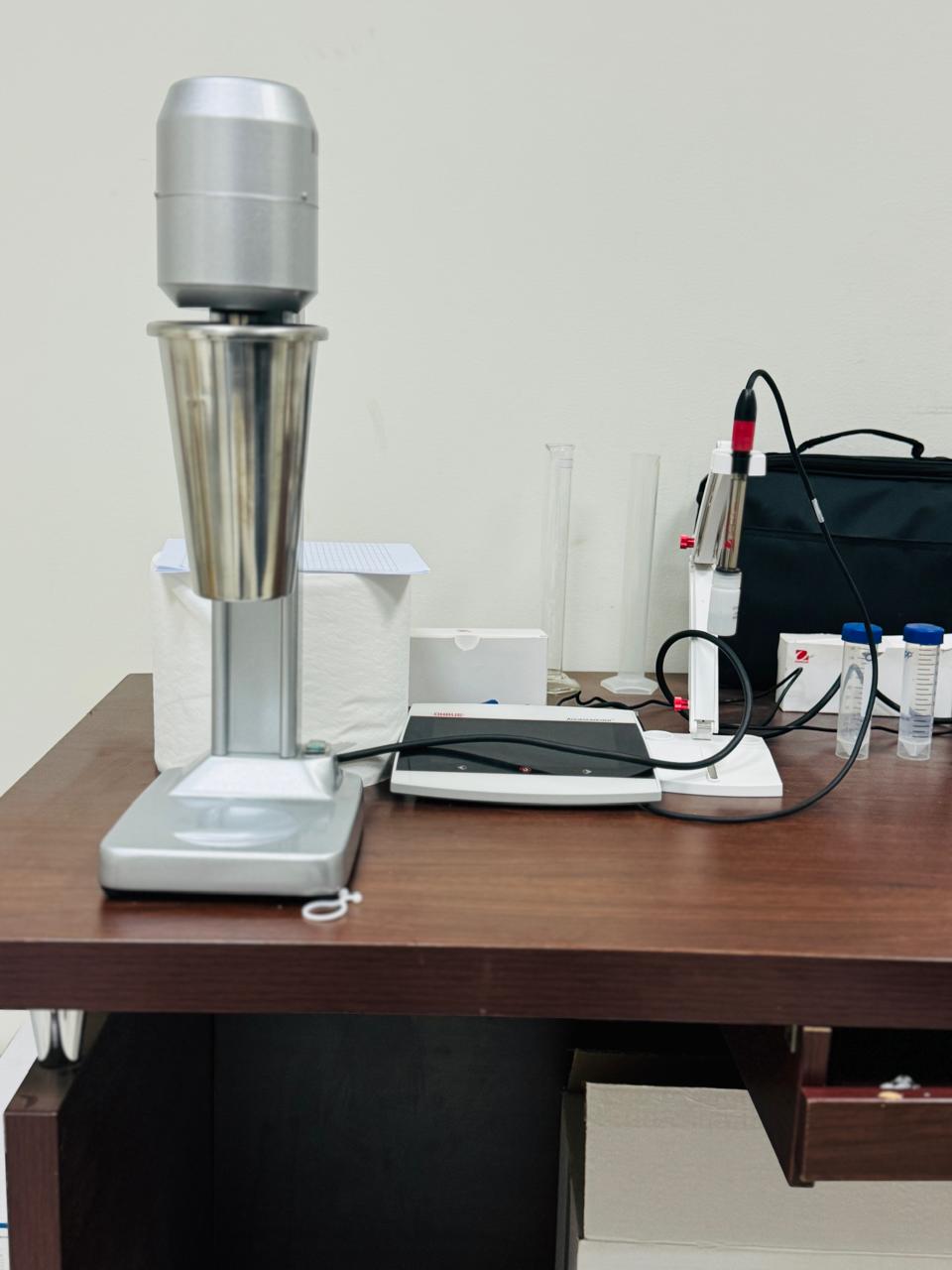
  **
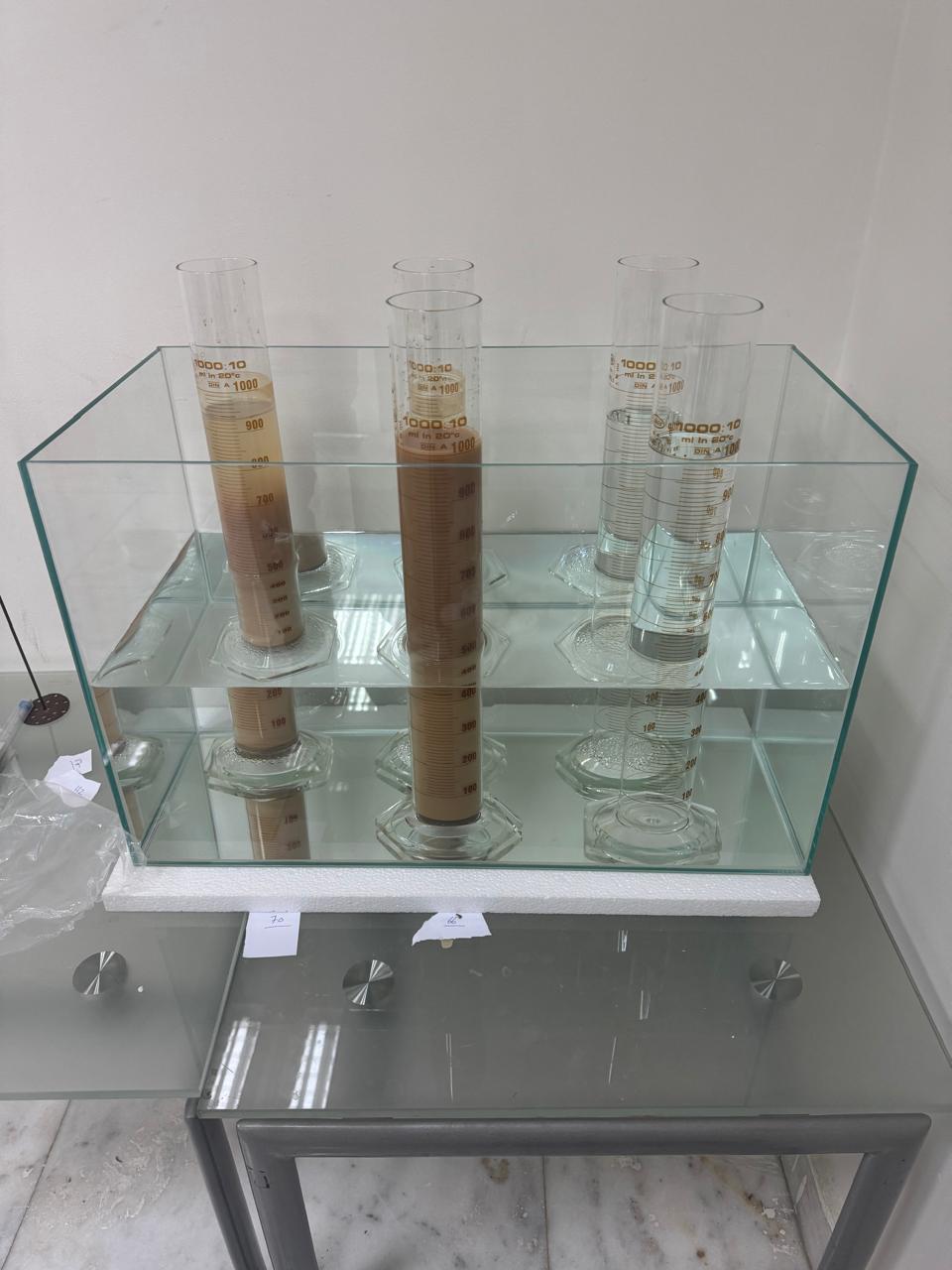
**

**Soil texture analysis (Hydrometer method)**
