# Supplementary material for: Monitoring land degradation by soil salinity using Sentinel-2 satellite data and GIS techniques: A case study of Sabkhat Ghuwaymid, Saudi Arabia
Source: PLoS One. 2026 May 13;21(5):e0348799. doi: 10.1371/journal.pone.0348799 (PMC13170892; doi:10.1371/journal.pone.0348799)

**Statistical Analysis**

**Stepwise multiple linear regression analysis of the Developed model**

| **Descriptive Statistics** | | | |
| --- | --- | --- | --- |
|  | Mean | Std. Deviation | N |
| EC | 45.03150 | 23.925981 | 72 |
| B1 | .111308 | .1028536 | 72 |
| B2 | .142079 | .0953886 | 72 |
| B3 | .215001 | .0810319 | 72 |
| B4 | .299386 | .0676920 | 72 |
| B5 | .333358 | .0669280 | 72 |
| B6 | .336439 | .0632761 | 72 |
| B7 | .344114 | .0616093 | 72 |
| B8 | .338978 | .0592035 | 72 |
| B9 | .350432 | .0600239 | 72 |
| B11 | .378486 | .0724844 | 72 |
| B12 | .301021 | .0783641 | 72 |
| SI_1_ | .433948 | .2096643 | 72 |
| SI_2_ | -.423959 | .2057675 | 72 |
| SI_3_ | -.423959 | .2057675 | 72 |
| SI_4_ | .187844 | .0974218 | 72 |
| SI_5_ | 1.291469 | .1692629 | 72 |
| NDSI | -.066572 | .0334489 | 72 |
| SBI | .076439 | .0409191 | 72 |
| INT1 | .256777 | .0722294 | 72 |
| INT2 | .426477 | .0998484 | 72 |
| MI | -.049743 | .0917561 | 72 |
| DEM | 584.95486 | .644851 | 72 |

| **Correlations** |
| --- |

**Correlations**

|  | | Pearson Correlation | Sig. (1-tailed) |
| --- | --- | --- | --- |
| **EC** | EC | 1.000 | . |
|  | B1 | .422 | .000 |
|  | B2 | .372 | .001 |
|  | B3 | .303 | .005 |
|  | B4 | .172 | .074 |
|  | B5 | .163 | .086 |
|  | B6 | .144 | .114 |
|  | B7 | .121 | .156 |
|  | B8 | .099 | .203 |
|  | B9 | .065 | .294 |
|  | B11 | -.373 | .001 |
|  | B12 | -.556 | .000 |
|  | SI_1_ | .446 | .000 |
|  | SI_2_ | .423 | .000 |
|  | SI_3_ | .423 | .000 |
|  | SI_4_ | .391 | .000 |
|  | SI_5_ | .684 | .000 |
|  | NDSI | .305 | .005 |
|  | SBI | .265 | .012 |
|  | INT1 | .267 | .012 |
|  | INT2 | .228 | .027 |
|  | MI | .496 | .000 |
|  | DEM | -.681 | .000 |

| **Model Summary** | | | | | | | | | |
| --- | --- | --- | --- | --- | --- | --- | --- | --- | --- |
| Model | R | R Square | Adjusted R Square | Std. Error of the Estimate | Change Statistics | | | | |
|  |  |  |  |  | R Square Change | F Change | df1 | df2 | Sig. F Change |
| 1 | .684^a^ | .468 | .461 | 17.569297 | .468 | 61.671 | 1 | 70 | .000 |
| 2 | .755^b^ | .570 | .558 | 15.912802 | .102 | 16.332 | 1 | 69 | .000 |
| 3 | .779^c^ | .606 | .589 | 15.338304 | .036 | 6.266 | 1 | 68 | .015 |
| a. Predictors: (Constant), SI_5_ | | | | | | | | | |
| b. Predictors: (Constant), SI_5_, SI_2_ | | | | | | | | | |
| c. Predictors: (Constant), SI_5_, SI_2_, DEM | | | | | | | | | |
| d. Dependent Variable: EC | | | | | | | | | |

| **ANOVA** | | | | | | |
| --- | --- | --- | --- | --- | --- | --- |
| Model | | Sum of Squares | df | Mean Square | F | Sig. |
| 1 | Regression | 19036.517 | 1 | 19036.517 | 61.671 | .000^b^ |
|  | Residual | 21607.614 | 70 | 308.680 |  |  |
|  | Total | 40644.131 | 71 |  |  |  |
| 2 | Regression | 23172.141 | 2 | 11586.070 | 45.755 | .000^c^ |
|  | Residual | 17471.990 | 69 | 253.217 |  |  |
|  | Total | 40644.131 | 71 |  |  |  |
| 3 | Regression | 24646.208 | 3 | 8215.403 | 34.920 | .000^d^ |
|  | Residual | 15997.923 | 68 | 235.264 |  |  |
|  | Total | 40644.131 | 71 |  |  |  |
| a. Dependent Variable: EC | | | | | | |
| b. Predictors: (Constant), SI_5_ | | | | | | |
| c. Predictors: (Constant), SI_5_, SI_2_ | | | | | | |
| d. Predictors: (Constant), SI_5_, SI_2_, DEM | | | | | | |

| **Coefficients** | | | | | | | | |  |
| --- | --- | --- | --- | --- | --- | --- | --- | --- | --- |
| Model | | Unstandardized Coefficients | | Standardized Coefficients | t | Sig. | Collinearity Statistics | | |
|  |  | B | Std. Error | Beta |  |  | Tolerance | VIF | |
| 1 | (Constant) | -79.904 | 16.043 |  | -4.981 | .000 |  |  | |
|  | SI_5_ | 96.739 | 12.319 | .684 | 7.853 | .000 | 1.000 | 1.000 | |
| 2 | (Constant) | -54.671 | 15.815 |  | -3.457 | .001 |  |  | |
|  | SI_5_ | 89.532 | 11.299 | .633 | 7.924 | .000 | .975 | 1.026 | |
|  | SI_2_ | 37.562 | 9.294 | .323 | 4.041 | .000 | .975 | 1.026 | |
| 3 | (Constant) | 6694.389 | 2696.305 |  | 2.483 | .016 |  |  | |
|  | SI_5_ | 55.942 | 17.282 | .396 | 3.237 | .002 | .387 | 2.582 | |
|  | SI_2_ | 34.413 | 9.047 | .296 | 3.804 | .000 | .956 | 1.046 | |
|  | DEM | -11.466 | 4.581 | -.309 | -2.503 | .015 | .380 | 2.633 | |
| a. Dependent Variable: EC | | | | | | | | |  |

| **Collinearity Diagnostics** | | | | | | | |
| --- | --- | --- | --- | --- | --- | --- | --- |
| Model | Dimension | Eigenvalue | Condition Index | Variance Proportions | | | |
|  |  |  |  | (Constant) | SI_5_ | SI_2_ | DEM |
| 1 | 1 | 1.992 | 1.000 | .00 | .00 |  |  |
|  | 2 | .008 | 15.432 | 1.00 | 1.00 |  |  |
| 2 | 1 | 2.852 | 1.000 | .00 | .00 | .02 |  |
|  | 2 | .140 | 4.510 | .01 | .02 | .89 |  |
|  | 3 | .008 | 19.304 | .99 | .97 | .09 |  |
| 3 | 1 | 3.837 | 1.000 | .00 | .00 | .01 | .00 |
|  | 2 | .153 | 5.011 | .00 | .00 | .90 | .00 |
|  | 3 | .011 | 19.104 | .00 | .39 | .07 | .00 |
|  | 4 | 2.261E-7 | 4119.069 | 1.00 | .61 | .02 | 1.00 |
| a. Dependent Variable: EC | | | | | | | |

| **Residuals Statistics** | | | | | |
| --- | --- | --- | --- | --- | --- |
|  | Minimum | Maximum | Mean | Std. Deviation | N |
| Predicted Value | 23.79142 | 93.14166 | 45.03150 | 18.631417 | 72 |
| Std. Predicted Value | -1.140 | 2.582 | .000 | 1.000 | 72 |
| Standard Error of Predicted Value | 2.008 | 10.318 | 3.305 | 1.476 | 72 |
| Adjusted Predicted Value | 24.33944 | 108.53016 | 45.39191 | 19.544708 | 72 |
| Residual | -34.591309 | 45.579033 | .000000 | 15.010758 | 72 |
| Std. Residual | -2.255 | 2.972 | .000 | .979 | 72 |
| Stud. Residual | -2.506 | 3.130 | -.010 | 1.036 | 72 |
| Deleted Residual | -51.953495 | 50.573467 | -.360408 | 17.012697 | 72 |
| Stud. Deleted Residual | -2.611 | 3.358 | -.007 | 1.065 | 72 |
| Mahal. Distance | .231 | 31.141 | 2.958 | 4.479 | 72 |
| Cook's Distance | .000 | 1.298 | .039 | .160 | 72 |
| Centered Leverage Value | .003 | .439 | .042 | .063 | 72 |
| a. Dependent Variable: EC | | | | | |


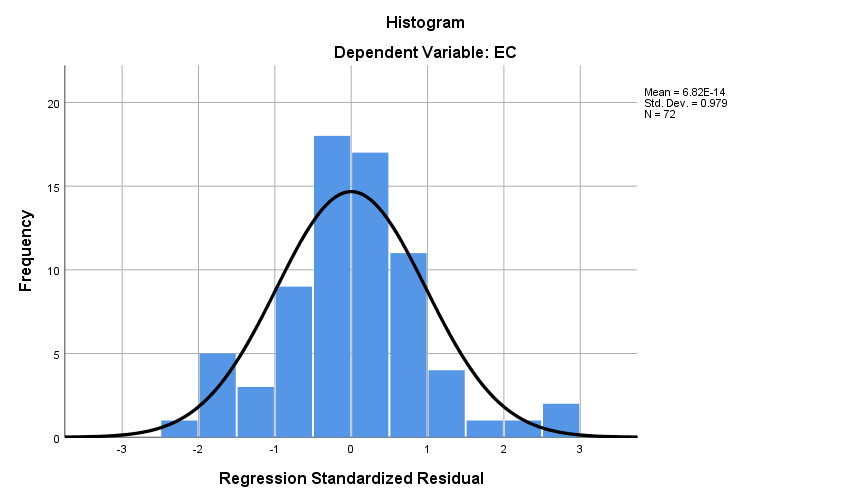


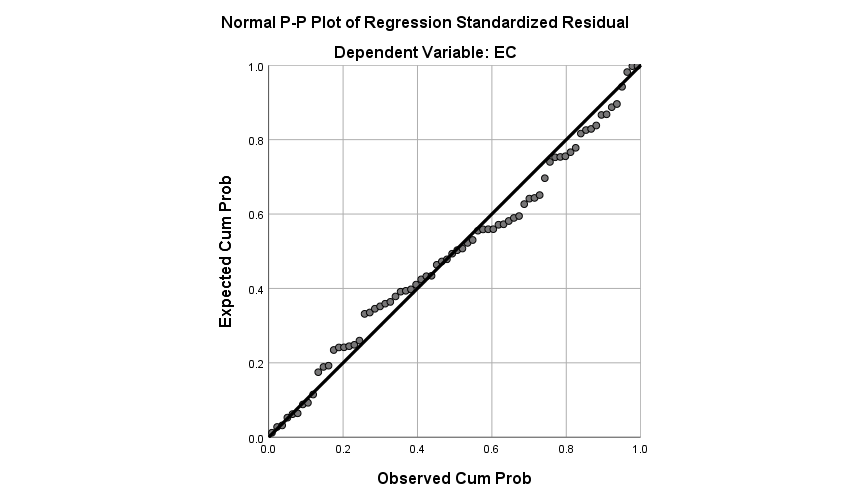


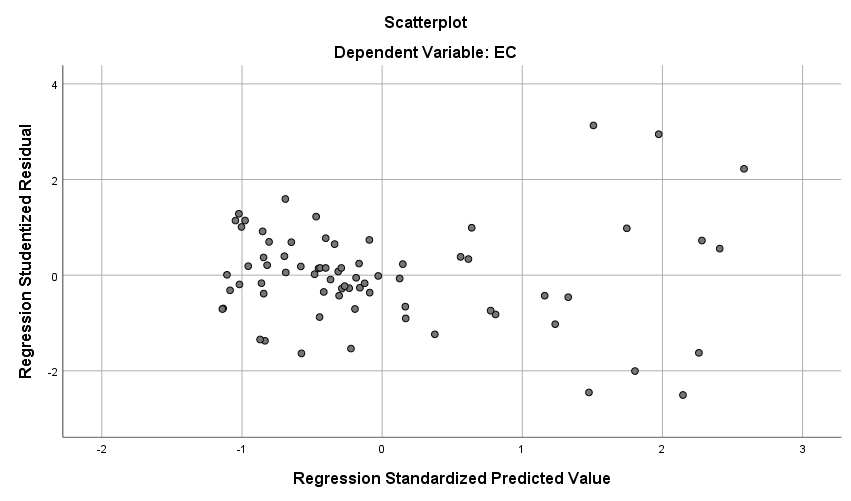


**Model:**


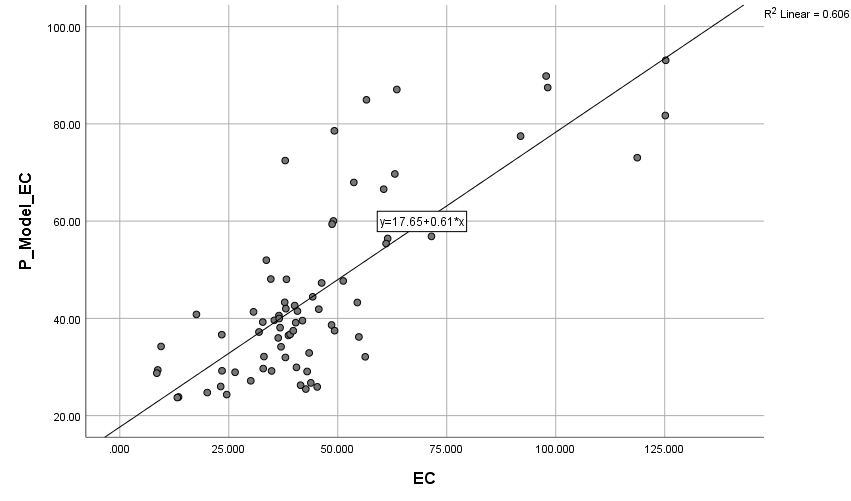


**Validation:**


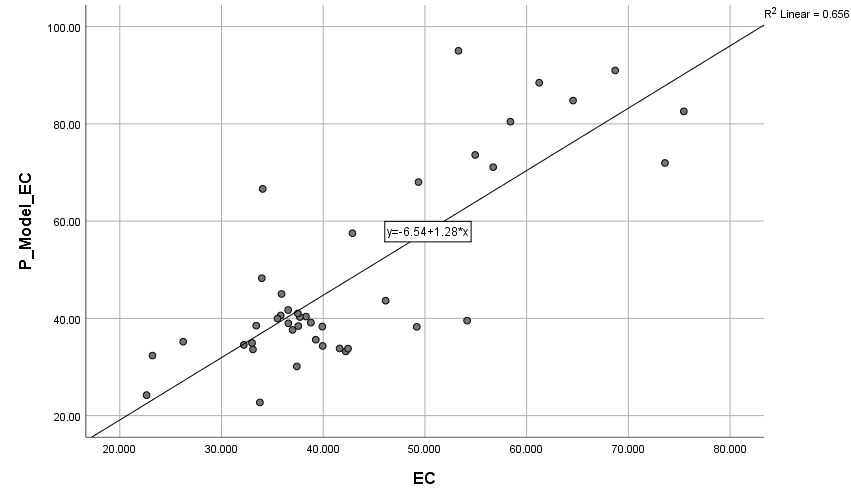

Supplement: S2 Tables — (DOCX) [file pone.0348799.s003.docx]
